# Exploration of genes and identification of evolutionary evidence in adeno-associated viruses

**DOI:** 10.71150/jm.2511016

**Published:** 2026-02-28

**Authors:** Chanhee Lee, Jihong Min, Somin Lim, Anyeseu Park, Seokjin Kwak, Soyeon Hwang, Sooyeon Park, Yong-Suk Jang, Se-Yeoun Cha, Sung-Gook Cho, Jeong Yoon Lee

**Affiliations:** 1 Laboratory of Viromics and Evolution, Korea Zoonosis Research Institute, Jeonbuk National University, Iksan 54531, Republic of Korea; 2 Department of Veterinary and Animal Science, Jeonbuk National University, Iksan 54596, Republic of Korea; 3 Department of Molecular Biology, Jeonbuk National University, Jeonju 54896, Republic of Korea; 4 College of Veterinary Medicine and Center for Avian Disease, Jeonbuk National University, Iksan 54596, Republic of Korea; 5 Department of Biotechnology, Korea National University of Transportation, Jeungpyeong 27909, Republic of Korea

**Keywords:** adeno-associated virus, rep gene, cap gene, virus evolution, proteotyping

## Abstract

Adeno-associated virus (AAV) commonly infects humans and non-human primates, generally inducing mild or even asymptomatic outcomes. AAVs have been shaped and diversified by evolutionary pressures, resulting in the identification of 13 serotypes thus far. Each serotype of AAV exhibits distinct tissue tropisms, targeting various organs, including the lung, central nervous system (CNS), liver, and skeletal muscle, thereby establishing AAVs as widely utilized vectors for therapeutic gene delivery. Bioinformatics analysis of specific viruses enables the inference of evolutionary patterns and offers valuable insights for predicting the emergence of novel viruses. While DNA sequence-based analysis has effectively facilitated the observation of mutation patterns accumulating within specific genes, it often provides limited insight into the actual impact of these mutations on proteins, the fundamental functional units. Utilizing proteotyping, an amino acid sequence-based comparative analysis, we identified hypervariable regions (HVR) within the AAV Cap gene and revealed concentrated evolutionary pressures in serotypes 4, 5, 11, and 12. Furthermore, we found that AAV-5 proteins exhibited considerable amino acid sequence divergence compared to those of other serotypes. Despite divergence, all AAV-5 proteins maintained a noticeable structural similarity to their counterparts in other serotypes. Our findings provide sequence-based insights into the evolutionary processes of AAV, facilitating the efficient identification of novel viruses.

## Introduction

Adeno-associated virus (AAV) is a small virus that can infect humans and certain non-human primates. AAV is a naked virus and has about 4.7 kb of positive single-stranded DNA (ssDNA) genome in a capsid (Naso et al., 2017; Zinn and Vandenberghe, 2014). AAV is classified in the Dependoparvovirus genus in the Parvoviridae family. In order to replicate, AAV must co-infect with other viruses, such as adenovirus and herpes virus (Geoffroy and Salvetti, 2005). The AAV genome is composed of two genes, named Rep and Cap; the Rep gene is associated with viral replication and integration of the host genome, while the Cap gene is associated with construction of the virion structure (Chiorini et al., 1996; Van Vliet et al., 2008). The Rep gene contains four transcription units (Rep78, Rep68, Rep52, and Rep40), while the Cap gene encodes three structural proteins (VP1, VP2, and VP3) and two accessory proteins (AAP and MAAP) (Chen, 2008; Galibert et al., 2021; Sonntag et al., 2011). The proteins encoded by both of these genes within the AAV genome interact with components of the host and helper virus during their life cycle. Due to its inability to replicate without a helper virus and its stability, which enables infection without causing disease, AAV is being investigated as a gene delivery vector for therapeutic applications.

To date, 13 types of AAV that primarily infect humans have been naturally identified. These viruses exhibit distinct amino acid sequences in their capsid proteins, which contribute to their different tropisms and tissue-specific targeting capabilities (Issa et al., 2023). For successful gene therapy, it is crucial to match the target tissue with a virus type that exhibits the appropriate tropism. To achieve optimal expression of the transgene delivered by AAV vectors, most studies primarily utilize AAV2, which is known for its superior replication efficiency (Kimura et al., 2019; Wang et al., 2024). Subsequently, through the development of recombinant vectors and modifications of these serotypes, a variety of recombinant AAVs continue to be developed (Balakrishnan and Jayandharan, 2014; Gao et al., 2005). To maximize the efficacy of gene therapy using AAVs, it is essential to understand the characteristic differences among the various types and to select the type that is most suitable for the research objectives.

Sequence similarity analysis is a widely used bioinformatics approach for identifying the accumulation of mutations within DNA sequences (Neira et al., 2017; Park et al., 2015). However, because events such as synonymous mutations, which do not result in changes to the amino acid sequence, occur frequently, not all variations at the DNA level impact the functional unit of proteins. For this reason, to understand the molecular evolution patterns of different virus types, it is important to analyze variations at the protein level rather than at the DNA level. Previous studies have shown that differences in genetic alteration, which are not observable at the DNA level, can be detected at the protein level through a widely used method known as proteotyping, which involves comparative analysis of amino acid sequences (Obenauer et al., 2006; Robinson et al., 2013). In previous studies, we discovered that multiple genes within specific viruses were involved in evolution based on proteotyping analysis (Harman et al., 2020; Lee et al., 2024). In summary, proteotyping analysis can elucidate detailed evolutionary patterns and provide insights into the potential emergence of new viruses.

In this study, we confirmed the evolutionary direction through DNA and protein level analyses of all genes within the AAV genome. Two hypervariable regions were identified in the Cap gene, demonstrating their evolutionary significance. Employing bioinformatics tools, this study conducted a comparative analysis of whole AAV genomes, elucidating overall patterns of genetic variation and their evolutionary implications.

## Materials and Methods

### DNA sequence analysis

The whole genome sequence information of 13 AAVs was collected from NCBI (https://www.ncbi.nlm.nih.gov/) and used in the DNA analysis (Table 1). Using the muscle algorithm in MEGA 11 software, the DNA sequence was aligned for Molecular Evolutionary Genetics Analysis (http://www.megasoftware.net). The Maximum Likelihood method was employed to construct the phylogenetic tree in MEGA 11 software, which was then converted to a Newick file and visualized in iTOL (https://itol.embl.de/). Full-length sequences of 13 AAV genomes were compared using SimPlot 3.5.1 software with a 200 bp window and a 20 bp step size.[Table t2-jm-2511016]

### Proteotyping

The amino acid sequences of 9 proteins encoded by the Rep and Cap genes for 13 AAVs were aligned using the Muscle algorithm, and each phylogenetic tree was created with MEGA 11 software using the maximum likelihood method. Using the SnapGene viewer program (https://www.snapgene.com/snapgene-viewer), the consensus sequence was established from the aligned amino acid sequences, applying a threshold of more than 50%. In the data, consensus sequences were marked in white, while gaps were indicated in black. If the amino acid sequence between the two types showed a divergence exceeding 10% relative to the total amino acid sequence, they were classified as different proteotypes. A unique color was designated for each amino acid.

### dN/dS analysis

DNA sequences of hypervariable regions (HVRs) within the Cap gene were aligned based on their corresponding amino acid sequences using the MUSCLE algorithm. These sequences were then subjected to back-translation after being converted into amino acid sequences via the TranslatorX program, and the resulting aligned sequences were saved in FASTA format. The estimation of pairwise Non-synonymous/Synonymous (dN/dS) ratios was performed using the Tamura-Nei model via MEGA11 software. Finally, the comparative dN/dS ratios among AAV serotypes were visualized as a heatmap using RStudio software.

### Three-dimensional protein structure prediction

Three-dimensional protein structures for individual genes of AAV2 and AAV5 were predicted based on their respective amino acid sequences using AlphaFold3 (https://alphafoldserver.com). The predicted structures were visualized using PyMOL 3.1 (https://www.pymol.org/). pLDDT values were utilized to assess the confidence and accuracy of predicted protein 3D structures (Abramson et al., 2024).

## Results

### Analysis of the DNA level in the whole genomes of 13 AAVs

**Fig. 1.**
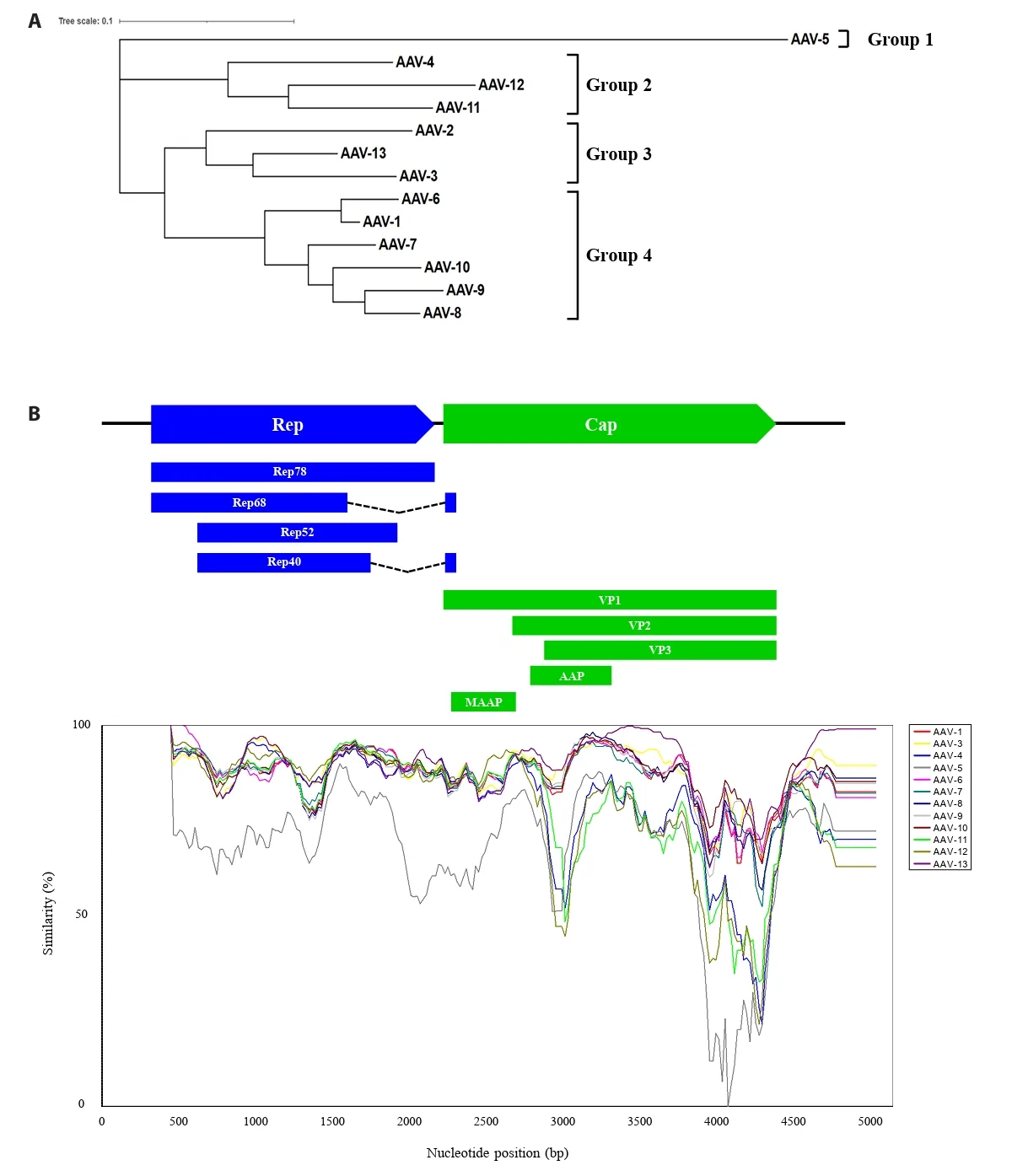
Phylogenetic tree and DNA sequence similarity analysis of the whole genome sequence of 13 AAVs. (A) A phylogenetic tree analysis of the AAV whole genome was performed using the maximum likelihood (ML) method in the MEGA 11 program (http://www.megasoftware.net/). (B) Similarity of 13 AAVs was analyzed with window 200 bp, step whole genome sequence of 13 AAVs was analyzed with a window of 200 bp, a step of 20 bp, Kimura (2-parameter), and AAV-2 serves as the query sequence. The X-axis and Y-axis represent nucleotide positions and percentage of sequence similarity, respectively. Positions of each gene based on AAV-2 are shown at the top of the graph.

To explore the evolutionary lineage among 13 distinct AAVs, we elucidated the phylogenetic relationship and genetic diversity. The whole-genome sequences of the 13 AAVs required for the analysis were obtained from NCBI and aligned using the MUSCLE algorithm in the MEGA11 program, which was subsequently formatted in FASTA format. Phylogenetic analysis was also conducted using the MEGA program, which classified AAV5 into a distinct group, while the other AAV serotypes grouped into three clusters (Fig. 1A). To investigate the distribution patterns of mutations across the genome, a SimPlot assay was conducted on 13 AAV isolates (Fig. 1B). AAV2 was designated as the query sequence, and each AAV type was assigned a specific color for differentiation. Analysis of the Rep gene region revealed generally high similarity, with the identification of two regions exhibiting relatively lower sequence similarity. Notably, the Cap gene region contained two distinct highly variable sections, located within the AAP gene and the common region of VP1, VP2, and VP3, respectively. These findings indicate the accumulation of mutations within the AAV genome, suggesting their potential involvement in the genetic variability of the virus.

### Genome-wide screening of AAVs via proteotyping-based protein profiling

**Fig. 2.**
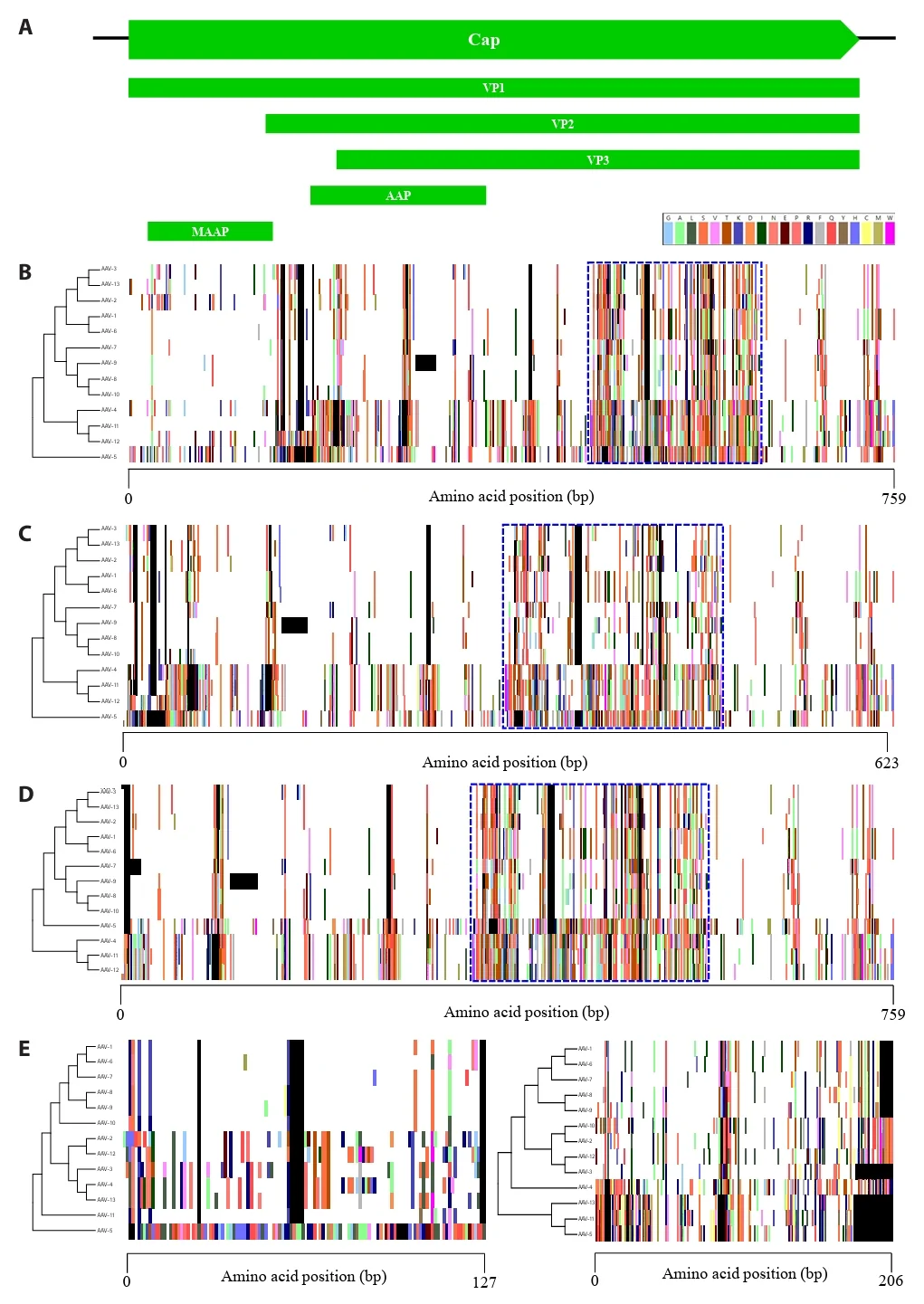
Proteotyping analysis based on the amino acid sequence of 5 transcription units in the Cap gene. (A) Map of the Cap gene. (B) VP1. (C) VP2. (D) VP3. (E) MAAP and AAP. The maximum likelihood phylogenetic tree for each protein (Left) is shown. The color assigned to each amino acid is indicated top. The blank and consensus sequences are represented in black and white, respectively. The hypervariable regions of VP1, VP2, and VP3 amino acid sequences are highlighted with blue dashed boxes.

Analysis at the DNA level identified various mutation regions throughout the adeno-associated virus (AAV) genome. However, DNA-level analysis by itself presents challenges in detecting non-synonymous point mutations or frameshifts resulting from insertions and deletions. Therefore, to determine the actual evolutionary impact of these regions, protein-level analysis is indispensable in addition to DNA-level investigation. We performed a comparative analysis of nine proteins encoded by the Rep and Cap gene regions using proteotyping, a biological analysis tool based on amino acid sequences. In the Rep gene region, four distinct transcription units are present, which encode proteins expressed through alternative splicing (Fig. S1A). Rep68 and Rep40, which are formed by the joining of two distinct exons, were grouped into two proteotypes, while Rep78 and Rep52 were classified into five proteotypes (Fig. S1B–S1E). The Cap gene encodes three major structural proteins that initiate translation from distinct start codons, along with two accessory proteins (Fig. 2A). VP1, VP2, and VP3, which consist of the capsid, were grouped into 10, 9, and 10 proteotypes, respectively (Fig. 2B–2D). Furthermore, we found substantial amino acid sequence diversity, which we consequently designated as hypervariable regions (HVRs), aligning well with the highly variable sections identified by SimPlot analysis (Fig. 1B). Especially, MAAP, corresponding to the unique N-terminus of VP1, exhibited the lowest number of proteotypes, with eight identified, whereas AAP exhibited the highest number of proteotypes, comprising 12 distinct groups (Fig. 2E). Consistently, amino acid sequence analysis revealed high variability accumulated within the Cap gene, mirroring the findings from DNA sequence analysis.

### Evidence of positive selection in hypervariable regions of AAV Cap genes across serotypes

**Fig. 3.**
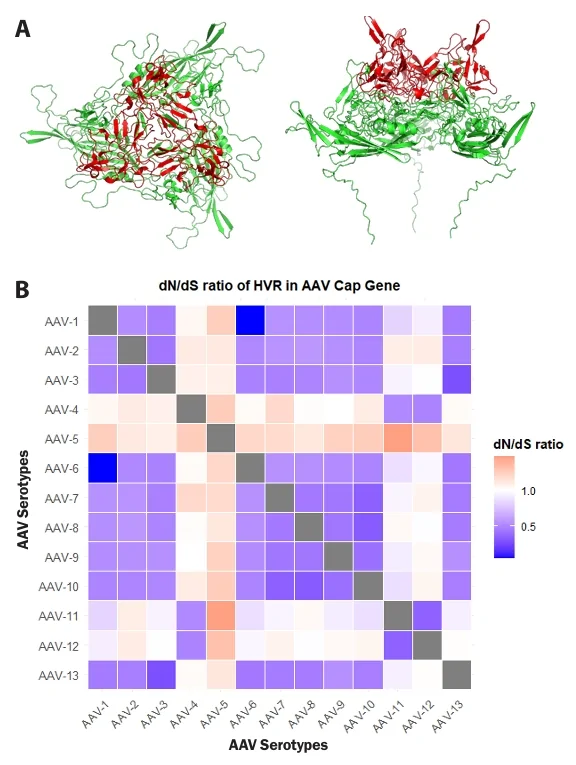
Selection pressure of hypervariable regions (HVRs) in the Cap gene. (A) Predicted structure of the AAV2 VP3 trimer using AlphaFold 3, visualized with PyMOL. Residues corresponding to HVRs within the Cap gene are highlighted in red. The left panel shows a top view, and the right panel shows a side view. (B) Pairwise dN/dS ratios for 13 AAV serotypes were analyzed using MEGA 11 software. The comparison of dN/dS ratios between each serotype was visualized as a heatmap using RStudio software. The X and Y axes represent AAV serotypes. Scale factors for each value are indicated on the right of the Fig.

To investigate whether the identified highly variable regions within the Cap gene significantly contributed to AAV evolution, additional analyses were performed. To elucidate the spatial arrangement of amino acid residues within these HVRs, the structure of VP3, the primary constituent of the AAV capsid, was visualized as a trimer. The visualized VP3 trimer revealed that HVR residues are predominantly located in surface-exposed regions of the capsid (Fig. 3A). This positional characteristic suggests a potential evolutionary link to evasion from the host immune system and adaptation for interaction with novel host cell receptors (Finlay and McFadden, 2006; Wu et al., 2012).

Pairwise dN/dS ratios were calculated between 13 AAV serotypes to evaluate the evolutionary pressures acting on the hypervariable regions (HVRs) of their Cap genes, and these ratios were visualized as a lower-triangle heatmap (Fig. 3B). Overall, the majority of pairwise comparisons involving AAV Cap gene HVRs exhibited dN/dS values less than 1, consistent with negative selection. However, distinct instances of elevated dN/dS ratios were also observed. For example, pairwise comparisons, such as those between AAV1 and AAV2, or AAV6 and AAV13, showed dN/dS ratios significantly below 1, indicating strong purifying selection in these HVRs. This observation supports the functional conservation of the Cap HVRs, allowing for minimal changes in amino acid sequence. Notably, pairwise comparisons involving AAV4 and AAV5 consistently revealed dN/dS ratios greater than 1 in their Cap HVRs when compared to other serotypes, indicative of positive selection. These dN/dS patterns revealed heterogeneous selective pressures acting on HVRs of the AAV Cap gene, encompassing both strong purifying selection and localized positive selection, thereby contributing to the observed serotype diversity. Collectively, these results underscore the crucial role of the designated HVRs in AAV evolution.

### Protein foldability of AAV-5's distinct amino acid sequences

**Fig. 4.**
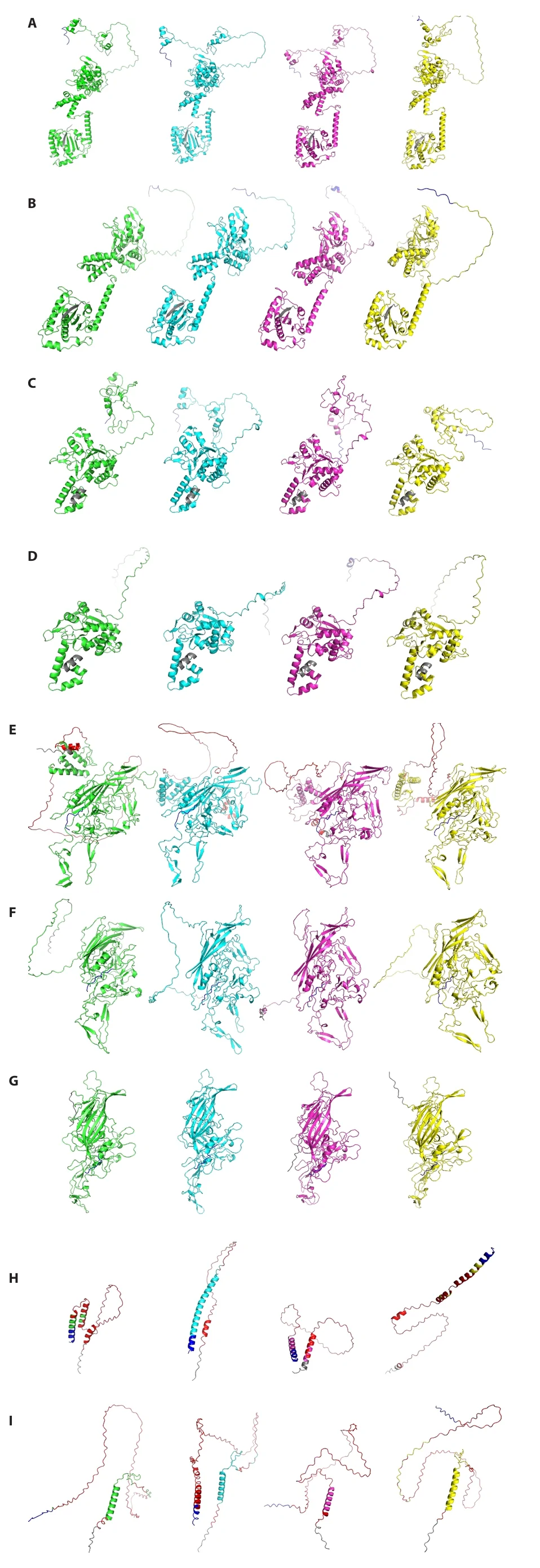
Comparative analysis of predicted 3D protein structures of AAV1, AAV2, AAV4, and AAV5. (A) Rep78. (B) Rep68. (C) Rep52. (D) Rep40. (E) VP1. (F) VP2. (G) VP3. (H) MAAP. (I) AAP. AAV1, AAV2, AAV4, and AAV5 are represented by green, cyan, magenta, and yellow, respectively. For each protein, the N- and C-terminal 10 amino acids are indicated by gray and blue, respectively. Red indicates regions of the predicted structure with a pLDDT value below 70%.

Given the observed high amino acid sequence variability of AAV5 proteins compared to other serotypes (Figs. 2, S1, and S3), comparative three-dimensional (3D) structure prediction was further investigated to determine whether this sequence divergence translates into significant structural differences for four Rep proteins and five Cap proteins derived from AAV5 (Fig. 4). Based on genomic analysis (Fig. 1A), AAV1, AAV2, and AAV4 were selected as representative serotypes from distinct phylogenetic groups. Homologous proteins from these serotypes were subsequently collected for a comprehensive comparative analysis. Remarkably, while their amino acid sequences showed significant divergence, most predicted 3D structures, with the notable exceptions of VP1, MAAP, and AAP, maintained considerable structural similarity. Specifically, the predicted VP1 structure demonstrated alpha-helical orientation similarities exclusively with AAV4 (phylogenetic Group 2) and AAV1 (phylogenetic Group 4). In contrast to VP1, MAAP, and AAP exhibited distinct differences in their predicted structures, including variations in the number and length of α-helices. The confidence of these predicted protein structures for VP1, MAAP, and AAP, as indicated by pLDDT values, is presented in Fig. 4. We observed that MAAP and AAP consistently showed pLDDT values below 70% across the majority of their protein sequences across all investigated types. Conversely, VP1 proteins displayed low pLDDT values specifically within their N-terminal regions. Genomic sequence analysis further revealed that this low-confidence N-terminal segment of VP1 directly overlaps with the coding regions of both MAAP and AAP (Fig. S4). Critically, these comparative 3D structure predictions, particularly those involving AAV5, consistently revealed that AAV proteins largely preserve their overall conserved tertiary architecture, notwithstanding substantial amino acid sequence variations.

## Discussion

The proteotyping analysis, which integrates genomic and structural predictions, provides crucial insights into the evolutionary strategies of naturally occurring adeno-associated viruses (AAVs). It explains how they maintain functional structures despite changes in their genetic sequences. We propose that the significant variability observed within the AAV Cap gene (Fig. 1) reflects advantageous mutations, which are vital for the virus’s survival. AAV5 exemplifies this; six of its nine proteins exhibit high structural similarity, despite considerable amino acid differences, compared to other serotypes. Consistent with our previous in silico analyses of adenovirus E4 (Lee et al., 2024; Park et al., 2024), we found similar mutation accumulation in the non-structural proteins of the Rep gene. Interestingly, even with high DNA sequence similarity, these proteins showed distinct proteotypes (Fig. S1). This clearly highlights that protein-level analysis is essential for revealing amino acid differences not visible at the DNA level, making it critical for a comprehensive understanding of AAV evolution and adaptation.

Earlier research identified specific variable regions (VRs) in VP1, VP2, and VP3 by comparing amino acid sequences among different serotypes (Govindasamy et al., 2006; Nam et al., 2007). These analyses mapped regions of high sequence conservation alongside areas exhibiting concentrated amino acid variations. Furthermore, X-ray crystallography and cryo-EM revealed the structural details of these nine variable regions, which typically present as surface-exposed loops or protrusions on the capsid (Gurda et al., 2013; Lerch et al., 2010). These variations likely reflect evolutionary strategies that enable the virus to acquire functional traits critical for survival, including tropism, antigenicity, and host immune evasion (Finlay and McFadden, 2006; Havlik et al., 2020; Nisanov et al., 2025). Our proteotyping results identified HVRs that notably included regions corresponding to VRs IV through VIII. Interestingly, proteotyping conducted solely on these HVR sequences demonstrated that all AAV types, except AAV-1 and AAV-6, which displayed identical proteotypes across their full genomes, exhibited distinct proteotyping profiles (Fig. S2). This finding suggests that comparative analysis of the designated HVR sequences within the Cap gene alone could be sufficient for distinguishing novel wild-type AAV serotypes. Moreover, this approach holds promise for predicting the specific characteristics of capsids modified with new patterns, offering valuable implications for AAV vector engineering and diagnostics. To further investigate whether HVR sequences were shaped by selective pressure, we performed dN/dS analysis and confirmed instances of positive selection in specific AAV types via a heat map. Particularly, AAV-4 and AAV-5, which are evolutionarily close on the phylogenetic tree, generally exhibited dN/dS values greater than 1 when compared to other types. AAV-11 and AAV-12 also showed dN/dS values exceeding 1 in comparison with certain types. This suggests that HVR variations gave advantageous functional characteristics to capsid proteins, promoting evolutionary fixation and underscoring a crucial role in viral survival.

While dN/dS analysis offers valuable insights into gene sequence selection pressures, its interpretation is inherently complicated by methodological limitations, especially in rapidly evolving systems such as AAV capsids. However, only comparing the endpoints can lead to misinterpretations. This is because factors such as unobserved intermediate mutations, sequence saturation, pronounced time-dependent effects, and the masking of non-synonymous changes by subsequent or reverting substitutions can collectively lead to an underestimate of the true evolutionary dynamics (Moses and Durbin, 2009; Mugal et al., 2020; Rocha et al., 2006). Therefore, to achieve a comprehensive understanding of AAV evolution, it is crucial to move beyond static dN/dS snapshots by incorporating time-dependent models and accounting for multiple substitutions over evolutionary distances. Furthermore, to gain a more dynamic perspective and address these analytical limitations more directly, exploring the utility of long-term in vitro passaging experiments with AAV would be highly valuable. Such studies could facilitate the direct observation of mutation accumulation and subsequent phenotypic shifts, thereby providing crucial empirical data to complement and validate computational dN/dS analyses.

Based on phylogenetic analysis at the genomic level, AAV-5 was found to be phylogenetically distinct from other AAV types and was placed in a separate phylogenetic group. Furthermore, amino acid sequence analysis revealed a generally high degree of variability across all proteins, including those encoded by both the Rep and Cap genes. Despite this considerable sequence variation, previous studies that elucidated the structure of AAV-5 showed that its fundamental core scaffold structure remained largely similar to that of AAV-2, although functionally critical surface structures significantly differed (Walters et al., 2004). Given the conserved protein folding essential for their function, we hypothesized that AAV-5 proteins would share similar tertiary structures, even with large amino acid sequence divergence. To investigate this, we predicted the monomeric structures of nine AAV-5 proteins and assessed their structural similarity to those of other AAV types. Interestingly, six of the AAV-5 proteins were predicted to adopt similar tertiary structures when compared to the predicted protein structures of other AAV types from distinct phylogenetic groups. The observed lower structural similarity of VP1 is likely attributable to its larger size as the open reading frame (ORF) of the entire Cap gene, which thus accommodates a greater extent of amino acid variations. Nevertheless, the overall protein scaffold appears to be maintained, suggesting a conservation of its fundamental architecture despite the high sequence plasticity. Moreover, MAAP and AAP showed less predicted structural similarity compared to other proteins. It is hypothesized that this is due to their relatively shorter sequences and greater inherent flexibility (Galibert et al., 2021; Maurer et al., 2018). These findings collectively suggest that the monomeric units of AAV-5 proteins can maintain similar overall structures despite displaying high amino acid sequence variability compared to other AAV types. This structural conservation presumably enables the formation of functional AAV virions and facilitates the essential functions required for the viral life cycle. Thus, AAV-5 clearly illustrates how a virus can evolve to both maintain its unique genetic makeup and acquire beneficial adaptations for host survival.

These findings underscore the profound evolutionary plasticity of protein folding. Despite the substantial amino acid sequence variability observed across all AAV-5 proteins, including Rep and Cap, when compared to other AAV types, AAV capsids, in particular, maintain a stable, core tertiary structure even with significant amino acid sequence divergence. This aspect is particularly relevant for HVRs of AAV, where residues consistently display elevated mutational tolerance (Guo et al., 2004). Specifically, AAV HVRs frequently accommodate amino acid substitutions without severely disrupting the overall protein fold or assembly. This suggests that while evolution exerts strong selective pressure for stable protein folding, it also facilitates subtle modulations to folding pathways or the acquisition of novel functions through specific amino acid substitutions in non-essential regions, thereby optimizing adaptive processes (Jemth, 2025; Vila, 2022, 2023). To fully elucidate these complex processes, experimental approaches are required that extend beyond current computational predictions. Furthermore, to fully understand the dynamic relationship between sequence evolution, protein folding, and viral adaptation, long-term in vitro evolution experiments are essential. When combined with biophysical characterization of selected variants, such studies could provide invaluable experimental data illustrating how AAV effectively balances structural stability with functional adaptation under specific selective pressures.

Viral evolution research extends beyond academic curiosity, directly and significantly influencing human health and global socio-economic stability. As seen in pandemics caused by influenza, human immunodeficiency virus (HIV), and SARS-CoV-2, viruses continuously mutate (Fischer et al., 2021; Wille and Holmes, 2020). This constant change diminishes vaccine efficacy and promotes the development of drug resistance. By predicting and understanding these viral evolutionary pathways, we can establish proactive vaccine and therapeutic strategies against future variants. Gene therapy vectors, including adeno-associated viruses (AAVs), fundamentally utilize the evolved characteristics of natural viruses. Specifically, understanding AAV capsid evolution enables the development of strategies to optimize gene therapy vector performance, for instance, by enhancing tissue tropism or reducing immunogenicity. In summary, this study provides bioinformatics evidence for the evolutionary dynamics of wild-type AAVs, thereby contributing a foundational direction for further viral evolutionary research.

## Figures and Tables

**Table 1.** 13 AAV genome information was collected by NCBI. DNA analysis was conducted using the data from the table, and amino acid sequence information was subsequently collected

Types	NCBI accession No.	Genome length (bp)	Year published
AAV-1	NC_002077.1	4718	VRL 13-AUG-2018
AAV-2	NC_001401.2	4679	VRL 13-AUG-2018
AAV-3	NC_001729.1	4726	VRL 13-AUG-2018
AAV-4	NC_001829.1	4767	VRL 13-AUG-2018
AAV-5	NC_006152.1	4642	VRL 13-AUG-2018
AAV-6	AF028704.1	4683	VRL 12-JAN-1998
AAV-7	NC_006260.1	4721	VRL 13-AUG-2018
AAV-8	NC_006261.1	4393	VRL 13-AUG-2018
AAV-9	LQ870207.1	4385	PAT 19-SEP-2018
AAV-10	AY631965.1	4102	VRL 30-NOV-2004
AAV-11	AY631966.1	4087	VRL 30-NOV-2004
AAV-12	DQ813647.1	4213	VRL 20-FEB-2008
AAV-13	EU285562.1	4180	VRL 23-SEP-2008

**Table 2.** The proteotypes of the nine open reading frames (ORFs) across 13 AAV serotypes are indicated by numerical codes, with each proteotype distinctly color-coded. Rows are organized according to VP1, while columns follow the linear order of ORFs arranged from the 5′ to 3′ end of the AAV genome.

Types	Rep			
	Rep78	Rep68	Rep52	Rep40
AAV-3	3	1	4	1
AAV-13	3	1	2	1
AAV-2	2	1	3	1
AAV-1	1	1	1	1
AAV-6	1	1	1	1
AAV-7	1	1	1	1
AAV-9	1	1	1	1
AAV-8	1	1	1	1
AAV-10	1	1	1	1
AAV-4	3	1	2	1
AAV-11	1	1	1	1
AAV-12	4	1	3	1
AAV-5	5	2	5	2
Total proteotypes	5	2	5	2

**Table:** 

Types	Cap				
	VP1	VP2	VP3	MAAP	AAP
AAV-3	1	1	1	4	7
AAV-13	1	1	1	6	6
AAV-2	2	2	2	2	5
AAV-1	3	3	3	1	1
AAV-6	3	3	3	1	1
AAV-7	4	4	4	1	2
AAV-9	5	5	5	1	8
AAV-8	6	5	6	1	3
AAV-10	6	5	6	1	4
AAV-4	7	6	8	5	10
AAV-11	8	7	9	7	11
AAV-12	9	8	10	3	12
AAV-5	10	9	7	8	9
Total proteotypes	10	9	10	8	12

## Data Availability

The supporting data for this study can be obtained from the corresponding author upon reasonable request.

## Supplementary Information

Fig. S1. Proteotyping analysis based on the amino acid sequence of 4 transcription units in the Rep gene. (A) Map of the Rep gene. (B) Rep78. (C) Rep68. (D) Rep52. (E) Rep40. The maximum likelihood phylogenetic tree for each protein (Left) is shown. The color assigned to each amino acid is indicated top. The blank and consensus sequences are represented in black and white, respectively.

Fig. S2. Proteotyping analysis based on the amino acid sequence of the HVR in the Cap gene. The maximum likelihood phylogenetic tree for each protein (Left) is shown. The color assigned to each amino acid is indicated top. The blank and consensus sequences are represented in black and white, respectively. The proteotypes of HVR in the Cap gene (Right) are represented by numbers, and each proteotype is clearly distinguished by a specific color.

Fig. S3. Multiple Amino Acid Sequence Alignment for the Detection of Sequence Variation in AAV5. (A) VP1. (B) MAAP. (C) AAP. Amino acid sequence mutations of AAV5 within the consensus sequence are marked in red.

Fig. S4. Evaluation of the confidence of predicted protein 3D structures. Evaluation of the confidence of predicted protein 3D structures. The confidence in the predicted VP1, AAP, and MAAP protein structures is indicated by pLDDT values. Red color represents pLDDT values below 50%, and yellow denotes values ranging from 50% to less than 70%. (A) AAV1, (B) AAV2, (C) AAV4, (D) AAV5.
