# Supplementary material for: Exploration of genes and identification of evolutionary evidence in adeno-associated viruses
Source: J Microbiol. 2026 Feb 28;64(2):e2511016. doi: 10.71150/jm.2511016 (PMC13577106; doi:10.71150/jm.2511016)
Supplement: Supplementary file 1 [file jm-2511016-Supplementary-Fig-S2.pdf]

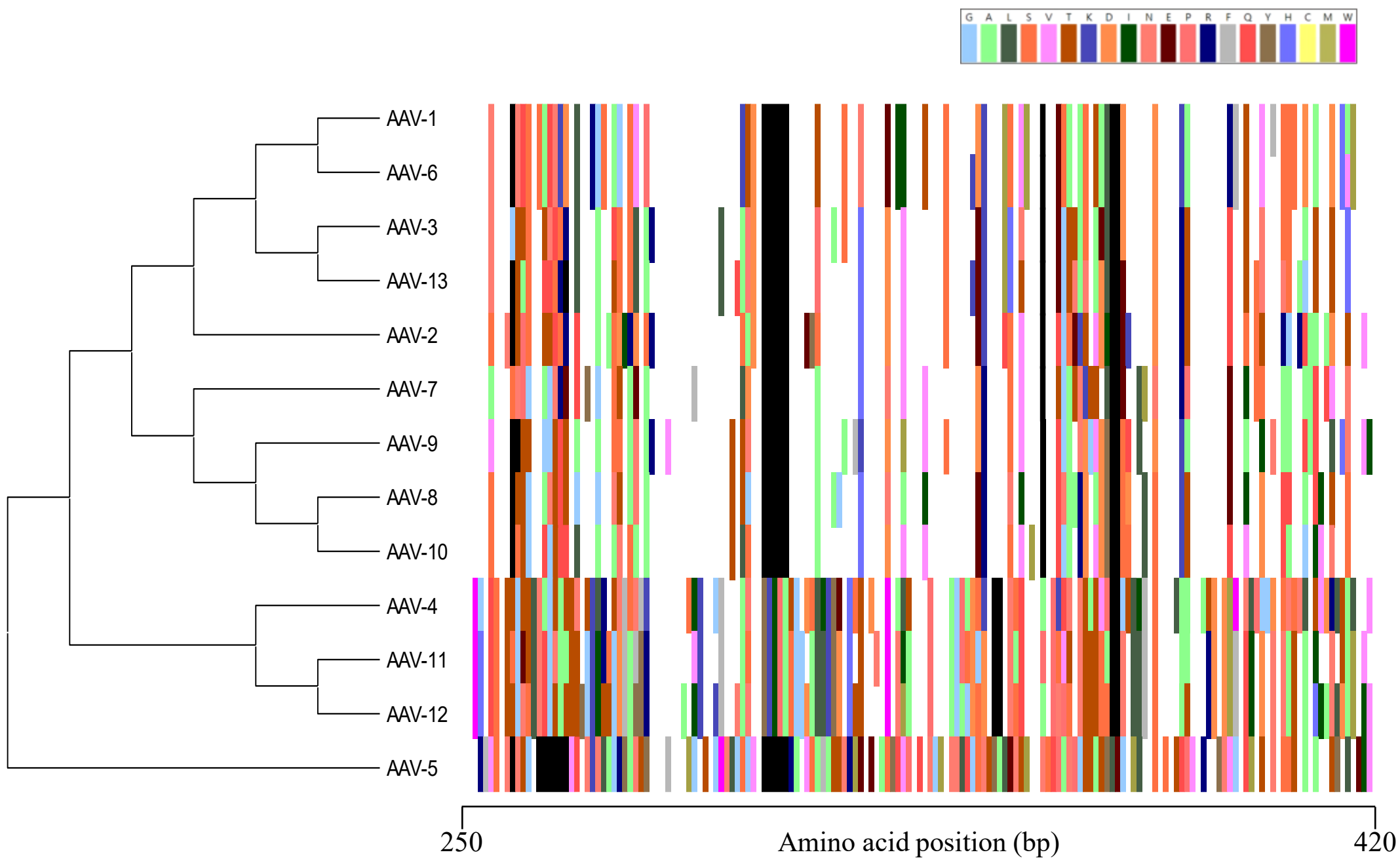

Types	HVR
AAV-1	1
AAV-6	1
AAV-3	2
AAV-13	3
AAV-2	4
AAV-7	5
AAV-9	6
AAV-8	7
AAV-10	8
AAV-4	9
AAV-11	10
AAV-12	11
AAV-5	12
Total proteotypes	12

Fig. S2. Proteotyping analysis based on the amino acid sequence of the HVR in the Cap gene. The maximum likelihood phylogenetic tree for each protein (Left) is shown. The color assigned to each amino acid is indicated top. The blank and consensus sequences are represented in black and white, respectively. The proteotypes of HVR in the Cap gene (Right) are represented by numbers, and each proteotype is clearly distinguished by a specific color.
