# Supplementary material for: Exploration of genes and identification of evolutionary evidence in adeno-associated viruses
Source: J Microbiol. 2026 Feb 28;64(2):e2511016. doi: 10.71150/jm.2511016 (PMC13577106; doi:10.71150/jm.2511016)
Supplement: Supplementary file 2 [file jm-2511016-Supplementary-Fig-S3.pdf]

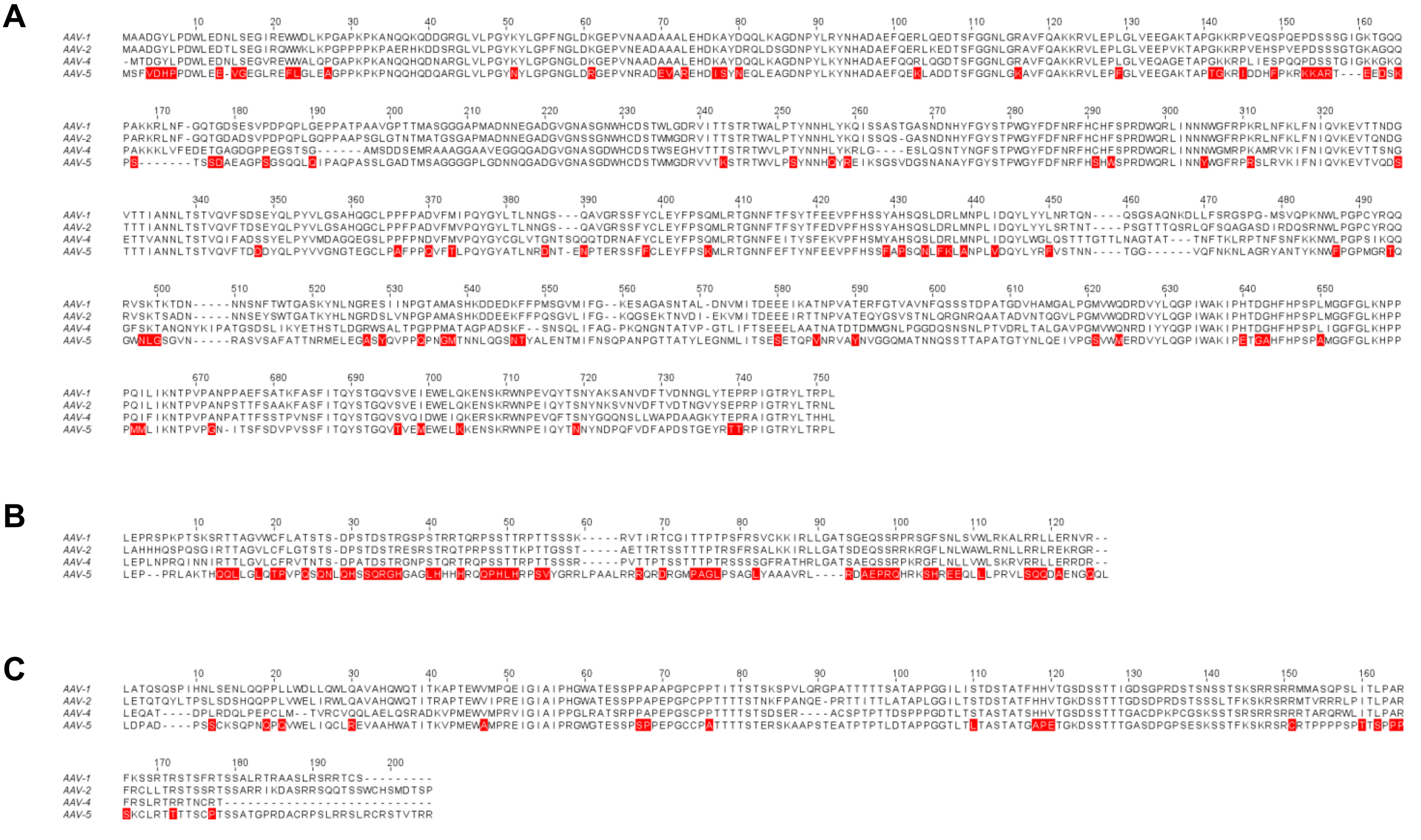

Fig. S3. Multiple Amino Acid Sequence Alignment for the Detection of Sequence Variation in AAV5. (A) VP1. (B) MAAP. (C) AAP. Amino acid sequence mutations of AAV5 within the consensus sequence are marked in red.
