# Supplementary material for: Exploration of genes and identification of evolutionary evidence in adeno-associated viruses
Source: J Microbiol. 2026 Feb 28;64(2):e2511016. doi: 10.71150/jm.2511016 (PMC13577106; doi:10.71150/jm.2511016)
Supplement: Supplementary file 3 [file jm-2511016-Supplementary-Fig-S4.pdf]

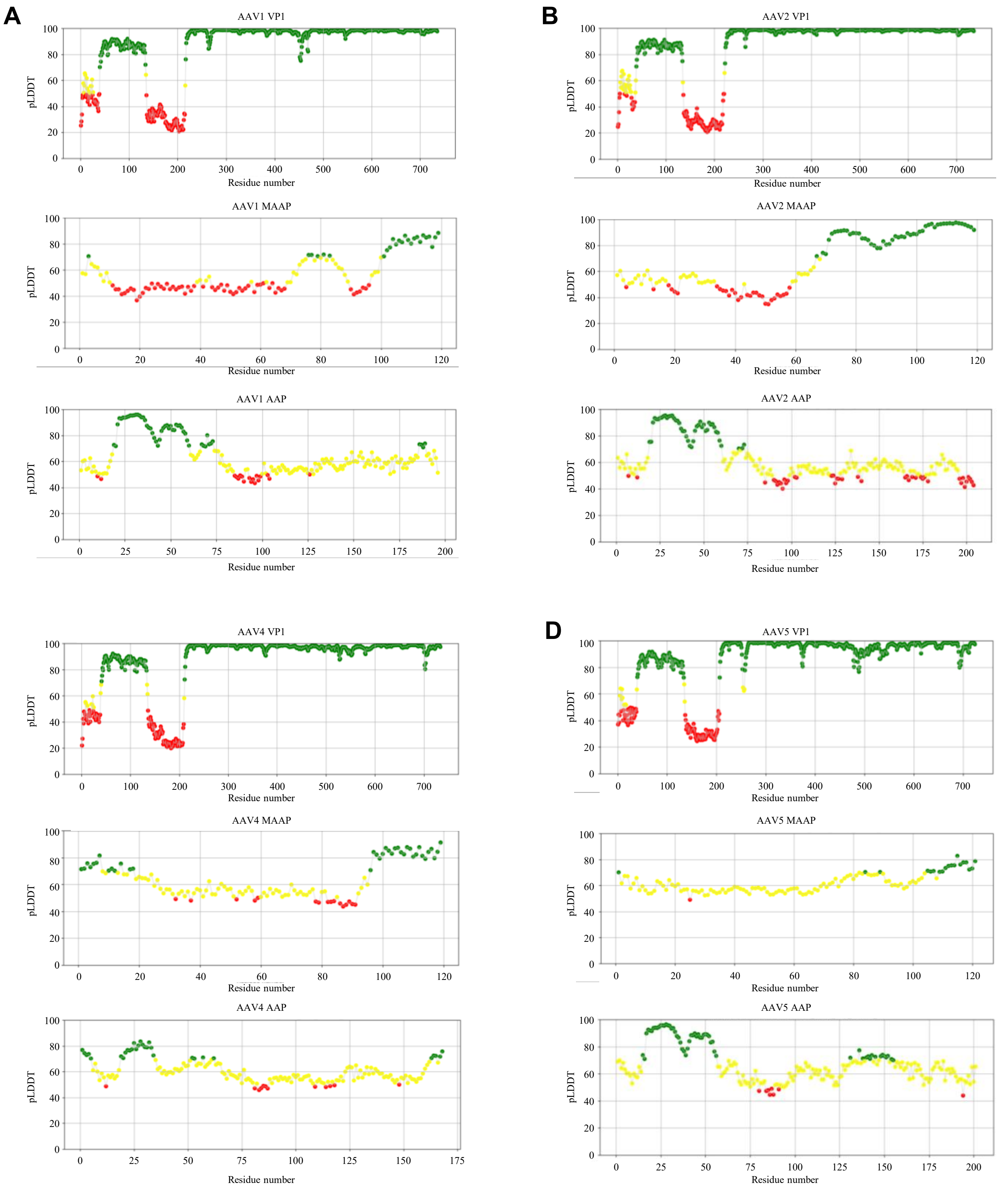

Fig. S4. Evaluation of the confidence of predicted protein 3D structures. Evaluation of the confidence of predicted protein 3D structures. The confidence in the predicted VP1, AAP, and MAAP protein structures is indicated by pLDDT values. Red color represents pLDDT values below 50%, and yellow denotes values ranging from 50% to less than 70%. (A) AAV 1, (B) AAV2, (C) AAV4, (D) AAV5.
